# Performance and clinical applicability of machine learning in liver computed tomography imaging: a systematic review

**DOI:** 10.1007/s00330-023-09609-w

**Published:** 2023-05-12

**Authors:** Keyur Radiya, Henrik Lykke Joakimsen, Karl Øyvind Mikalsen, Eirik Kjus Aahlin, Rolv-Ole Lindsetmo, Kim Erlend Mortensen

**Affiliations:** 1 Department of Gastroenterological Surgery at University Hospital of North Norway (UNN), Tromso, Norway; 2 Department of Clinical Medicine, UiT The Arctic University of Norway, Tromso, Norway; 3 Institute of Clinical Medicine, UiT The Arctic University of Norway, Tromso, Norway; 4 Centre for Clinical Artificial Intelligence (SPKI), University Hospital of North Norway, Tromso, Norway; 5 UiT Machine Learning Group, Department of Physics and Technology, UiT the Arctic University of Norway, Tromso, Norway; 6 Head Clinic of Surgery, Oncology and Women Health, University Hospital of North Norway, Tromso, Norway

**Keywords:** Liver neoplasms, Radiology, Tomography, X-ray computed, Artificial intelligence, Machine learning

## Abstract

**Objectives:**

Machine learning (ML) for medical imaging is emerging for several organs and image modalities. Our objectives were to provide clinicians with an overview of this field by answering the following questions: (1) How is ML applied in liver computed tomography (CT) imaging? (2) How well do ML systems perform in liver CT imaging? (3) What are the clinical applications of ML in liver CT imaging?

**Methods:**

A systematic review was carried out according to the guidelines from the PRISMA-P statement. The search string focused on studies containing content relating to artificial intelligence, liver, and computed tomography.

**Results:**

One hundred ninety-one studies were included in the study. ML was applied to CT liver imaging by image analysis without clinicians’ intervention in majority of studies while in newer studies the fusion of ML method with clinical intervention have been identified. Several were documented to perform very accurately on reliable but small data. Most models identified were deep learning-based, mainly using convolutional neural networks. Potentially many clinical applications of ML to CT liver imaging have been identified through our review including liver and its lesion segmentation and classification, segmentation of vascular structure inside the liver, fibrosis and cirrhosis staging, metastasis prediction, and evaluation of chemotherapy.

**Conclusion:**

Several studies attempted to provide transparent result of the model. To make the model convenient for a clinical application, prospective clinical validation studies are in urgent call. Computer scientists and engineers should seek to cooperate with health professionals to ensure this.

**Key Points:**

• *ML shows great potential for CT liver image tasks such as pixel-wise segmentation and classification of liver and liver lesions, fibrosis staging, metastasis prediction, and retrieval of relevant liver lesions from similar cases of other patients*.

• *Despite presenting the result is not standardized, many studies have attempted to provide transparent results to interpret the machine learning method performance in the literature*.

• *Prospective studies are in urgent call for clinical validation of ML method, preferably carried out by cooperation between clinicians and computer scientists*.

**Supplementary Information:**

The online version contains supplementary material available at 10.1007/s00330-023-09609-w.

## Introduction

For several tasks related to medical imaging, ML is emerging as a new reliable tool due to its high performance and a superior capacity to build complex models for making predictions [1]. More than 220 medical devices using ML have been approved in the USA and Europe [2]. This development has increased steadily since 2014. Today, ML software can be considered a medical device [3].

Computer tomography (CT) imaging plays an essential role in diagnostics and post-treatment follow-up in liver diseases [4]. Applying ML-based tools to CT images has shown promising results [5]. It has been tested theoretically for tasks including identification and segmentation of the liver, lesions, blood vessels, and bile ducts in the liver [6], quantification of liver tissue characteristics [7], evaluation of cancer treatment, and prediction of liver disease [8, 9].

A recently published systematic review and meta-analysis demonstrated the diagnostic accuracy of deep learning (DL) in ophthalmology, respiratory medicine, and breast surgery [10]. In addition, a limited literature review has been published in the subfield of ML applied to liver imaging [11–13]. However, the performance and clinical applicability of ML in liver imaging are not comprehensively addressed in the literature.

A search in PROSPERO—a database of prospectively registered systematic reviews in health and social care [14]—did not reveal any forthcoming publication in this rapidly developing field. We, therefore, conducted a systematic review from a clinical perspective.

This review aims to answer the following questions: (1) How is ML applied in CT liver imaging? (2) How well do ML systems perform in liver CT imaging? (3) What are the clinical applications of ML in liver CT imaging?

Some important part of this article is given in the electronic supplementary material due to length of the article.

### Methods

This systematic review was conducted in accordance with the guidelines for the “Preferred Reporting Items for Systematic Reviews and Meta-Analyses” extension for diagnostic accuracy studies statement [15]. A selection and retrieval of studies from the literature was done in accordance with Cochrane handbook for systematic review [16]. A search was conducted in Medline, EMBASE, and Web of Science and included studies published between January 1, 2011, and October 31, 2021. The search string consisted of exploded MeSH-terms, Emtree-terms, and free text to find all studies containing the terms “Artificial intelligence” AND “Computed tomography” AND “liver” (or containing all possible synonyms of all three) in the title, abstract, or keywords. The exact search string was given in the electronic supplementary material.

When considering study quality, we identified characteristics as important given in the electronic supplementary material. The suggested list is comprehensive, and studies might be quite informative with minimal risk of bias, without meeting all requirements [17]. Yet, if a study followed only few of the characteristics, it was not considered well-documented for clinical use.

## Results

**Fig. 1:**
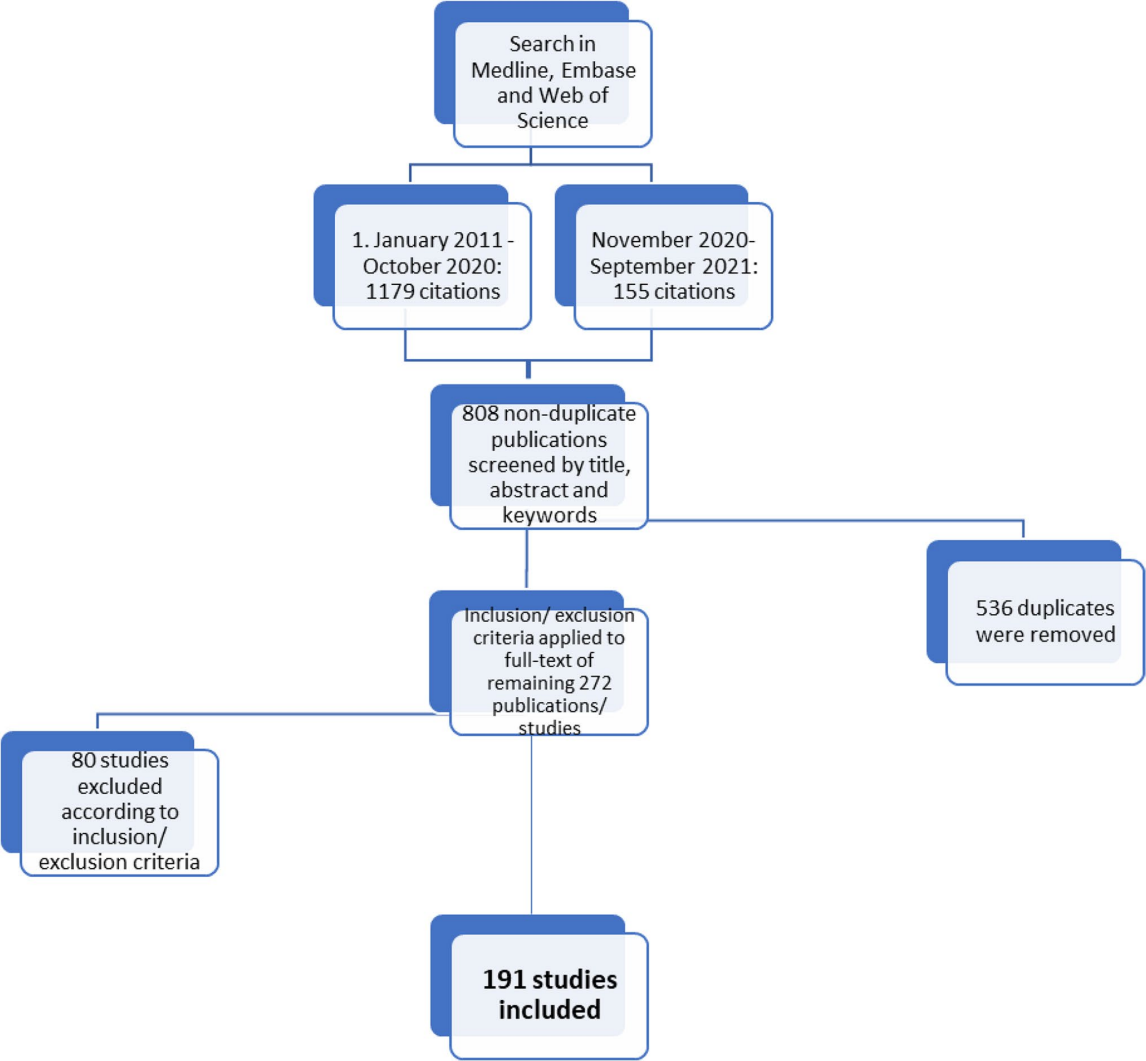
Prisma flow chart. Flow chart of systematically included 191 studies from 1334 identified studies from Medline, Embase, and Web of science

The search was conducted in two phases, one in October 2020 and one in October 2021. There were 191 studies included for review. The selection process is illustrated in the PRISMA flow diagram in Fig. 1 [18]. The selected studies are summarized in Table 1 and details given in the electronic supplementary material.

**Table 1:** Description of included studies with detail about included in group, document type A = article and PP = proceeding paper, type of journal – medical or non-medical, AI method used, amount of test set, external validity status, ML to clinician, using of publicly available datasets

Study	Group	Type of paper	Type of journal	Type of ML method used	Test Set	External validity	ML to clinician	Public dataset
Mubashir et al 2019 [19]	Liver segmentation	A	Non-medical	DBN-DNN	15 + 15 (2 open datasets)	No	No	Yes
Mubashir et al 2019 [19]	Liver segmentation	PP	Non-medical	CNN	5	No	No	Yes
Ahn et al 2020 [20]	Liver segmentation	A	Medical	3d U-net, DLA DeepLabV3	20 CT series and 60 CT series	Yes	Yes	Yes
Bhavya et al 2018 [21]	Liver segmentation	PP	Medical	Real AdaBoost classifier	70	Yes	No	Yes
Albishri 2019 [22]	Liver segmentation	PP	Non-medical	Cascade U-net	32 patient’s data (unclear about the total number of data)	No	No	No
Ali 2017 [23]	Liver segmentation	PP	Non-medical	SVM	50	No	Yes	Yes
Alirr 2020 [24]	Liver segmentation	A	Non-medical	U NET + level set	20	Yes	Yes	Yes
Astono 2018 [25]	Liver segmentation	PP	Non-medical	CNN-adjacent net	10 scans	Yes	No	Yes
Ben-Cohen 2016 [26]	Liver segmentation	PP	Non-medical	FCN-VGG 16-layer net	70 CT sets	Yes	No	Yes
Bevilacqua et al 2017 [27]	Liver segmentation	PP	Non-medical	ANN classifier by using mono-objective genetic algorithm (GA)	Not mentioned	Yes	No reference	No
Bhole 2011 [28]	Liver segmentation	PP	Non-medical	MRF	10 series of 10 patients	No	No	Yes
Budak et al 2020 [29]	Liver segmentation	A	Medical	CEDCNN	5 sets (589 slices)	No	No	Yes
Cai 2019 [30]	Liver segmentation	A	Medical	Adaptive scale-kernel fuzzy clustering models	They have created 3 model from different dataset, and the fourth model for fine tuning. Difficult to give in number of patients used in for training. As they have used transfer learning from one model to another model where adding some more data	Yes	No	Yes
Chen 2019 [31]	Liver segmentation	PP	Non-medical	MPNet, adversarial densely connected network and a deep FCNN	10	Yes	No	Yes
Chen et al 2019 [31]	Liver segmentation	A	Medical	Channel-U-net, a spatial channel wise convolutional neural network		Yes	No	Yes
Chlebus 2018 [32]	Liver segmentation	A	Non-medical	FCNN and object based postprocessing	Not mentioned	Yes	Yes	No
Choi et al 2018 [33]	Liver segmentation	A	Medical	CNN	150 images	Yes	Yes	No
Chung 2020 [34]	Liver segmentation	A	Non-medical	CENet	28 volumes	No	No	Yes
Danciu 2013 [35]	Liver segmentation	A	Medical	SVM	76 patients (20–40 images of the liver per patient)	No	Yes	Yes
Danciu 2012 [36]	Liver segmentation	PP	Non-medical	3D DCT and SVM	26,608 images of 70 CT scans from 40 patients	No	No	Yes
Delmoral 2019 [37]	Liver segmentation	PP	Non-medical	CNN	31CT	Yes	No	Yes
Dong 2020 [38]	Liver segmentation	A	Non-medical	HDCNN	50 patients, 1272 images	No	No	Yes
Dou et al 2016 [39]	Liver segmentation	A	Medical	3D deeply supervised network	5 patients for testing, 5 patients for validation	Yes	No	No
Guo 2019 [40]	Liver segmentation	A	Medical	FCNN	–	Yes	No reference	No
He et al 2016 [41]	Liver segmentation	A	Medical	Ada Boost guided active shape model	(1) 46 lesions for validation, 46 lesions for testing; (2) not specified	Yes	No	Yes
Heker 2019 [42]	Liver segmentation	PP	Non-medical	Cascade U-net	Not specified	Yes	No	No
Hu 2016 [43]	Liver segmentation	A	Medical	3D-CNN	10 patients	Yes	No	Yes
Huang et al 2012 [44]	Liver segmentation	PP	Non-medical	ELM	Not specified	Yes	No	No
Ji 2013 [45]	Liver segmentation	A	Non-medical	ACM	Not specified	Yes	No	No
Jiang 2018 [46]	Liver segmentation	A	Medical	Registration based organ positioning, FCMC, ELM, ACM	Not specified	No	No	Yes
Jiang 2019 [47]	Liver segmentation	A	Non-medical	3D FCN, AHCBlocks	12 images for validation, 1 for testing	Yes	No	Yes
Jin 2017 [48]	Liver segmentation	PP	Non-medical	FCN-U-net	25 patients for testing, 25 patients for validation	Yes	No	Yes
Kavur et al 2020 [49]	Liver segmentation	A	Medical	CNN	20 patients	No	No	Yes
Kumar 2016 [50]	Liver segmentation	A	Medical	Feedforward neural network	Not mentioned	No	No reference	No
Chung 2020 [34]	Liver segmentation	A	Non-medical	CNN (CENet)	150 images	No	No	No
Zheng et al 2019 [51]	Liver segmentation	PP	Non-medical	GAN + deep atlas prior	28 volumes	No	No	Yes
Zhang, Y. 2018 [52]	Liver segmentation	PP	Non-medical	FCN + CRF	5 scans/patients	No	No	Yes
Zhang, L. 2018 [53]	Liver segmentation	PP	Non-medical	U-net	76 patients (20–40 images of the liver per patient)	No	No	Yes
Xu 2019 [54]	Liver segmentation	PP	Non-medical	RES-U-Net, connected components analyzing and CRF	26,608 images of 70 CT scans from 40 patients	No	No	Yes
Xi 2020 [55]	Liver segmentation	A	Non-medical	Cascade U-RES-Net (CNN + Dlo + TL + GDL + GTL)	70 image sets	No	No	Yes
Xin 2020 [56]	Liver segmentation	A	Non-medical	CNN	32 patients, 643 slices containing lesions	No	No	No
Xia 2019 [57]	Liver segmentation	A	Non-medical	CNN Deep Adversarial Networks (DeepLab-v3) + weighted loss function	8800 images	No	No	Yes
Winkel et al 2020 [58]	Liver segmentation	A	Medical	DRL (CNN + RL)	20 sets, 6 sets per patient	No	Yes	No
Wang et al 2019 [59]	Liver segmentation	PP	Non-medical	CNN	28 patients	No	No	Yes
Tian 2019 [60]	Liver segmentation	PP	Non-medical	U-net (GLC-U-net, CNN)	50 patients, 1272 images	No	No	Yes
Tang 2017 [61]	Liver segmentation	PP	Non-medical	FCN (+ level set)	5 patients for testing, 5 patients for validation	No	No	No
Seo et al 2020 [62]	Liver segmentation	A	Non-medical	CNN (modified U-Net)	(1) validation: 5 patients; 2550 images; testing: 35 patients; 16,125 images; (2) 5 patients, 525 images	Yes	No	Yes
Selvi 2014 [63]	Liver segmentation	PP	Non-medical	High-order neural network	Not specified	No	No	No
Selvathi et al 2013 [64]	Liver segmentation	PP	Non-medical	ELM + FCMC	Not specified	No	No	No
Sayed 2016 [65]	Liver segmentation	PP	Non-medical	Fuzzy clustering + GWO (Liver and liver lesion segmentation); SVM (liver disease classification: benign/malignant)	Not provided	No	No	No
Sakboonyara 2019 [66]	Liver segmentation	PP	Non-medical	U-Net, 2D (CNN/ FCN)	5 images	No	No	Yes
K S et al 2018 [67]	Liver segmentation	PP	Non-medical	U-Net and 3D CRF	–	No	No	No
Raj 2016 [68]	Liver segmentation	PP	Non-medical	SVM	Not specified	No	No	No
Rafiei 2018 [69]	Liver segmentation	PP	Non-medical	FCN + CRF	10 patients	No	No	Yes
Qin et al 2018 [70]	Liver segmentation	A	Medical	CNN (SBBS-CNN, based on CifarNet)	Not specified	No	No	Yes
Ponnoprat et al 2020 [71]	Liver segmentation	A	Non-medical	U-Net for segmentation + CRF for post-processing + SVM for classification	17 patients, 2042 images	No	No	No
Ouhmich 2019 [72]	Liver segmentation	A	Medical	U-Net	Not specified	No	No	No
Ng et al 2020 [73]	Liver segmentation	A	Medical	Gaussian mixture model and U-Net	6 patients (fivefold cross validation)	No	No	No
Nayak et al 2019 [74]	Liver segmentation	A	Medical	Segmentation: region-growing; classification: SVM	Not specified	No	No	Yes
Mukherjee et al 2013 [75]	Liver segmentation	PP	Non-medical	SVM + PCA	Not specified	No	No	No
Morshid et al 2019 [76]	Liver segmentation	A	Medical	Segmentation: U-Net, 2D; prediction: RFC	Not specified	Yes	Yes	Yes
Mohagheghi and Foruzan 2020 [77]	Liver segmentation	A	Medical	U-Net	12 images for validation, 1 for testing	No	No	Yes
Mofrad 2014 [78]	Liver segmentation	A	Medical	Classification: SVM, k-NN	1 patient	No	No	No
Meng L 2020 [79]	Liver segmentation	A	Non-medical	TDP-CNN + CRF (post-processing)	25 patients for testing, 25 patients for validation	No	No	Yes
Luo and Li 2014 [80]	Liver segmentation	PP	Non-medical	SVM	1 image, 1 patient	No	No	Yes
Lu et al 2017 [81]	Liver segmentation	A	Medical	CNN + graph cut	SLiver07: 10 patients; 3D-IRCADb: 20 patients	Yes	Yes	Yes
Selvaraj 2013 [82]	Liver segmentation	PP	Non-medical	Lesion segmentation: FCM; feature selection: BPSO; classification: PNN	15 images	No	No	No
Li 2014 [83]	Liver segmentation	PP	Non-medical	PCA + ASM + k-NN	5 whole body scans, 5 abdominal contrast-enhanced scans	No	No	Yes
Li et al 2018 [84]	Liver segmentation	A	Non-medical	H-Dense U-Net	LiTS 2017: 70 patients; 3D-IRCADb: cross-validation	Yes	No	Yes
Liu et al 2019 [85]	Liver segmentation	A	Non-medical	U-Net + graph cut	20 patients	No	No	Yes
Linguraru et al 2012 [86]	Liver segmentation	A	Non-medical	SVM	LiTS 2008: 4 patients; SLiver07: 10 patients	Yes	No	Yes
Astono et al 2018 [25]	Liver segmentation	A	Non-medical	Adjacent Net	Validation: 2 patients; test: 2 × 10 patients	No	No	Yes
Afifi and Nakaguchi 2015 [87]	Liver segmentation	PP	Non-medical	MSCA + graph cut in detection	Not specified	No	No reference	No
Roth 2020 [88]	Liver segmentation	PP	Non-medical	U-net	70	Yes	No	Yes
Tran 2021 [89]	Liver segmentation	A	Non-medical	U-Net multilayer	30 scan (15 CT from each datasets)	Yes	No	Yes
Xu et al 2020 [90]	Liver segmentation	PP	Non-medical	pyramidal U-net	fourfold cross-validation	No	No reference	0
Yu et al 2021 [91]	Liver segmentation	A	Non-medical	DResU-Net	25	Yes	No	Yes
Zhang, Y et al 2021 [92]	Liver segmentation	A	Non-medical	RECIST NET	46	No	No	0
Zhang, Yao 2021 [92]	Liver segmentation	PP	Non-medical	CNN (deep attentive refinement network)	70	Yes	No	Yes
Ayalew 2021 [93]	Liver segmentation	A	Non-medical	U-net	392 images	No	No	Yes
Chen et al 2020 [94]	Liver segmentation	A	Medical	U-net	300 images	Yes	No	Yes
Chung 2021 [95]	Liver segmentation	A	Medical	CNN	80 patients	No	No	Yes
Elmenabawy et al 2020 [96]	Liver segmentation	PP	Non-medical	CDNN	33 patients	No	No	Yes
Fan 2020 [97]	Liver segmentation	A	Non-medical	U-net multi-scale attention net	70 patients	No	No	Yes
He et al 2021 [98]	Liver segmentation	A	Medical	U-net (3D RA-U-Net)	252 images, 63 patients	Yes	Yes	Yes
Kwon 2020 [99]	Liver segmentation	PP	Non-medical	U-net	70 patients	No	No	Yes
Lei 2020 [100]	Liver segmentation	PP	Non-medical	U-Net / V-Net	31 patients	No	No	Yes
Afifi 2015 [87]	Lesion detection	PP	Non-medical	Mean-shift segmentation algorithm	15 patients 169 lesions	No	No	No
Ali et al 2017 [23]	Lesion detection	PP	Non-medical	SVM	50	No	Yes	Yes
Ben-Cohen 2016 [26]	Lesion detection	PP	Non-medical	FCN-VGG 16 layer net	70 CT sets	Yes	No	Yes
Ben-Cohen 2018 [101]	Lesion detection	A	Non-medical	FCN8 net-VGG 16 layer net, and sparsity-based dictionary learning (localized patch level analysis usin superpixel sparse based classification	7 data sets	No	No	Yes
Bevilacqua et al 2017 [102]	Lesion detection	PP	Non-medical	ANN clssifier by using mono-objective GA	Not mentioned	Yes	No reference	No
Bevilacqua et al 2017 [102]	Lesion detection	PP	Non-medical	ANN clssifier by using MOGA	Not mentioned	No	No reference	No
Chen et al 2019 [103]	Lesion detection	PP	Non-medical	Dual-attention dilated residual network-weakly supervised localization	10 + 10 dataset from Sliver	No	No	Yes
Frid-Adar 2017 [104]	Lesion detection	PP	Non-medical	Multi-class patch based CNN system	(1) Validation: 5 patients, 2550 images, testing: 35 patients, 16,125 images; (2) 5 patients, 525 images	No	No	Yes
Furuzuki et al 2019 [105]	Lesion detection	PP	Non-medical	Faster R-CNN	Not specified	No	No	No
Gong et al 2019 [106]	Lesion detection	A	Medical	R-CNN, partial least square regression discriminant analysis model	5images	No	Yes	Yes
Huang et al 2013 [107]	Lesion detection	PP	Non-medical	Kernel-based ELM with classifier	17 patients, 2042 images	No	No	No
Jin 2017 [108]	Lesion detection	PP	Non-medical	CNN + ensemble learning	SLiver07: 10 patients; 3D-IRCADb: 20 patients	No	No	Yes
Jin 2015 [109]	Lesion detection	PP	Non-medical	Improved back propagation neural network	15 images	No	No	No
Kim 2019 [110]	Lesion detection	PP	Non-medical	Cycle-Consistent CNN	Not specified	No	No	No
Vivanti 2017 [111]	Lesion detection	A	Medical	CNN + RFC	Not specified	No	No	No
Tao et al 2019 [112]	Lesion detection	PP	Non-medical	FCN + RPN	∼ 5000 images for testing and ∼ 5000 images for validation	No	No	Yes
Liang et al 2019 [113]	Lesion detection	PP	Non-medical	CNN (recurrant with long short-term memory)	(1) validation: 175; test: 153; (2) validation: 175; test: 153	No	No	No
Lee 2018 [114]	Lesion detection	PP	Non-medical	SSD	fivefold cross-validation	No	No	No
Afifi 2015 [87]	Lesion detection	PP	Non-medical	MSCA (+ graph cut in detection)	Not specified	No	No reference	No
Yang et al 2021 [115]	Lesion detection	A	Non-medical	CNN	337	Yes	No reference	0
Zhou et al 2021 [116]	Lesion detection	A	Medical	CNN	1/4 of lesion was used for testset	No	No	0
Albishri 2019 [22]	Lesion segmentation	PP	Non-medical	Cascade U-net	32 patients data (unclear about the total number of data)	No	No	No
Alirr 2020 [24]	Lesion segmentation	A	Non-medical	U NET + level set	20	Yes	Yes	Yes
Almotairi 2020 [117]	Lesion segmentation	A	Non-medical	Modified Seg Net	20 CT from local hospital	No	No	Yes
Anter 2019 [118]	Lesion segmentation	A	Medical	Fast fuzzy C-means and adaptive watershed algorithm	30	Yes	No	Yes
Budak 2020 [29]	Lesion segmentation	A	Medical	CEDCNN	5 sets (589 slices)	No	No	Yes
Chen, L. 2019 [103]	Lesion segmentation	PP	Non-medical	MPNet, adversarial densely connected network and a deep FCNN	10	Yes	No	Yes
Chen, X. et al 2019 [119]	Lesion segmentation	PP	Non-medical	FED-Net	10 CT series	No	No	Yes
Chen, Y. et al 2019 [31]	Lesion segmentation	A	Medical	Channel-U -net		Yes	No	Yes
Chlebus 2018 [32]	Lesion segmentation	A	Non-medical	FCNN- and object-based postprocessing	Not mentioned	Yes	Yes	No
Delmoral 2019 [37]	Lesion segmentation	PP	Non-medical	CNN	31CT	Yes	No	Yes
Deng 2019 [120]	Lesion segmentation	A	Medical	Dynamic regulation to functional parameters over iterations using the 3D CNN	20 sets, 6 sets per patient	Yes	No	No
Dong 2020 [38]	Lesion segmentation	A	Non-medical	HDCNN	50 patients, 1272 images	No	No	Yes
Heker 2019 [42]	Lesion segmentation	PP	Non-medical	Cascade U-net	Not specified	Yes	No	No
Huang et al 2013 [107]	Lesion segmentation	PP	Non-medical	Kernel-based ELM with classifier	17 patients, 2042 images	No	No	No
Huang et al 2014 [121]	Lesion segmentation	PP	Non-medical	Random feature subspace ensemble–based ELM	6 patients (fivefold cross-validation)	No	No	No
Jiang 2018 [46]	Lesion segmentation	A	Medical	Registration based organ positioning, fuzzy C means clustering and ELM, ACM	Not specified	No	No	Yes
Jiang 2019 [47]	Lesion segmentation	A	Non-medical	3D FCN composed of multiple AHCBlocks	12 images for validation, 1 for testing	Yes	No	Yes
Kadoury 2015 [122]	Lesion segmentation	A	Medical	Grassmanian kernels and discriminant manifold, CRF	5 whole body scans, 5 abdominal contrast-enhanced scans	Yes	No	Yes
Almotairi 2020 [117]	Lesion segmentation	A	Non-medical	Modified SegNet	3 patients, 454 images for testing and 45 for validation	No	No	Yes
Zhou 2013 [123]	Lesion segmentation	PP	Non-medical	CNN	16 patients	Yes	No	No
Zhang, Yue et al 2020 [124]	Lesion segmentation	A	Non-medical	2D U-net + 3D FCN and unsupervised fuzzy c-means clustering	(1) 36 images; (2) 70 images	Yes	No	Yes
Zhang, Yi 2020 [125]	Lesion segmentation	A	Non-medical	CNN	(1) 9 sets for testing, 20 for verification/validation; (2) 5 for testing, 5 for verification	Yes	No	Yes
Zhang, Xing 2011 [126]	Lesion segmentation	PP	Non-medical	SVM + traditional feature extraction	Not specified	No	No	Yes
Xi 2020 [55]	Lesion segmentation	A	Non-medical	Cascade U-RES-Net (CNN + Dlo + TL + GDL + GTL)	70 image sets	No	No	Yes
Xin 2020 [56]	Lesion segmentation	A	Non-medical	CNN	32 patients, 643 slices containing lesions	No	No	No
Wu 2019 [127]	Lesion segmentation	PP	Non-medical	MW-U-net	15 patients, 100–135 images per patient	No	No	Yes
Wei et al 2019 [128]	Lesion segmentation	PP	Non-medical	CNN (HMMMNet)	(1) LiTS 2017: 26 patients; (2) decathlon: 70 (not specified in the article—found at medicaldecathlon.com)	No	No	Yes
Vorontsov et al 2018 [129]	Lesion segmentation	PP	Non-medical	CNN (FCN)	15 patients	No	No	Yes
Vorontsov et al 2017 [130]	Lesion segmentation	A	Non-medical	MLP	5 patients	No	No	No
Vivanti 2017 [111]	Lesion segmentation	A	Medical	CNN + RFC	Not specified	No	No	No
Vivanti 2018 [129]	Lesion segmentation	A	Non-medical	CNN (× 2: global and individual)	Not specified	No	No	No
Todoroki 2019 [131]	Lesion segmentation	PP	Non-medical	CNN	266,000, 282,000, and 215,000 patch images (tested once each)	No	No	No
Sun 2017 [132]	Lesion segmentation	PP	Non-medical	FCN	(1) 3D-IRCADb: 40 images; (2) JDRD: 36 images	Yes	No	Yes
Shimizu 2013 [133]	Lesion segmentation	A	Non-medical	U-Boost	Not specified	No	No	No
Seo 2020 [62]	Lesion segmentation	A	Non-medical	Modified U-Net	(1) Validation: 5 patients; 2550 images; testing: 35 patients, 16,125 images; (2) 5 patients, 525 images	Yes	No	Yes
Selvathi et al 2013 [64]	Lesion segmentation	PP	Non-medical	ELM + FCMC	Not specified	No	No	No
Sayed 2016 [65]	Lesion segmentation	PP	Non-medical	Segmentation: fuzzy clustering + GWO; classification: SVM	Not provided	No	No	No
Raj 2016 [68]	Lesion segmentation	PP	Non-medical	SVM	Not specified	No	No	No
Ouhmich 2019 [72]	Lesion segmentation	A	Medical	U-Net	Not specified	No	No	No
Morshid 2019 [76]	Lesion segmentation	A	Medical	Segmentation: U-Net; prediction: RFC	Not specified	Yes	Yes	Yes
Moawad et al 2020 [134]	Lesion segmentation	A	Medical	U-Net	Not specified	No	Yes	No
Meng et al 2020 [79]	Lesion segmentation	A	Non-medical	TDP-CNN + CRF	25 patients for testing, 25 patients for validation	No	No	Yes
Selvaraj 2013 [82]	Lesion segmentation	PP	Non-medical	Segmentaion: FCM; feature selection: BPSO; classification: PNN	15 images	No	No	No
Li et al 2018 [84]	Lesion segmentation	A	Non-medical	H-DenseU-Net	LiTS 2017: 70 patients; 3D-IRCADb: cross-validation	Yes	No	Yes
Radu et al 2020 [135]	Lesion segmentation	A	Medical	CNN	30 CT for testing	Internal	No	0
Roth 2020 [88]	Lesion segmentation	PP	Non-medical	U-net	70	External	No	Yes
Xin 2020 [56]	Lesion segmentation	A	Medical	CNN	643 slice for test	No	No	0
Tran 2021 [89]	Lesion segmentation	A	Non-medical	U-Net multilayer	30 scan (15 ct from each datasets)	Yes	No	Yes
Haq et al 2021 [136]	Lesion segmentation	PP	Non-medical	Resnet R-CNN	70	Yes	No	Yes
Yang et al 2021 [115]	Lesion segmentation	A	Non-medical	CNN	337	Yes	No reference	0
Yu et al 2021 [91]	Lesion segmentation	A	Non-medical	DResU-Net	25	Yes	No	Yes
Zhang, Yao 2021 [92]	Lesion segmentation	PP	Non-medical	CNN (deep attentive refinement network)	70	Yes	No	Yes
Anil 2021 [137]	Lesion segmentation	A	Non-medical	MDCN + FRN	NA	No	No	Yes
Aslam et al 2021 [138]	Lesion segmentation	A	Non-medical	ResU-Net	NA	No	No	Yes
Ayalew 2021 [93]	Lesion segmentation	A	Non-medical	U-net	392 images	No	No	Yes
Chen et al 2021 [94]	Lesion segmentation	A	Medical	U-net	300 images	Yes	No	Yes
Dey 2020 [139]	Lesion segmentation	PP	Non-medical	CNN	70 patients	No	No	Yes
Elmenabawy et al 2020 [96]	Lesion segmentation	PP	Non-medical	CDNN (conv-deconv neural net)	33 patients	No	No	Yes
Fan 2020 [97]	Lesion segmentation	A	Non-medical	U-net (multi-scale attention net)	70 patients	No	No	Yes
Hamard et al 2020 [140]	Lesion segmentation	A	Medical	NA (off the shelf product)	44	Yes	Yes	No
He et al 2021 [98]	Lesion segmentation	A	Medical	U-net (3D RA-U-Net)	252 images, 63 patients	Yes	Yes	Yes
Kwon 2020 [99]	Lesion segmentation	PP	Non-medical	U-net	70 patients	No	No	Yes
Adcock 2014 [18]	Classification	A	Non-medical	SVM-LibSVM (multidimensional scaling (CMDS)	Not mentioned	No	No	No
AmirHosseini 2019 [141]	Classification	A	Non-medical	Fuzzy inference system	7 patients for HCC segmentation, 20 patients for liver segmentation	No	No	Yes
Balagourouchetty et al 2020 [142]	Classification	A	Non-medical	GoogLeNet based Ensemble FCNet Classifier	Not mentioned exactly number but they have 10% data to test set and have used tenfold cross-validation	No	No	Yes
Bevilacqua et al 2017 [27]	Classification	PP	Non-medical	ANN classifier by using mono-objective genetic algorithm (GA)	Not mentioned	Yes	No reference	No
Cao et al 2020 [143]	Classification	A	Medical	Multiphase convolutional dense network	42CT (12 from local and 20 + 10 from Sliver07)	No	Yes	Yes
Chen et al 2019 [103]	Classification	PP	Non-medical	Dual-attention dilated residual network—weakly supervised localization	10 + 10 dataset from Sliver	No	No	Yes
Das 2019 [144]	Classification	A	Medical	Watershed Gaussian–based deep learning, DNN	32 patients, 643 slices containing lesions	No	No	No
Devi 2020 [145]	Classification	A	Non-medical	Region growing process for liver segmentation = > kernalized fuzzy C-means algorithm for lesion extraction, SVM-based classifier for classification of tumor	28 patients	No	No	Yes
Jiang 2013 [146]	Classification	A	Non-medical	SVM-multi instance learning	1 patient	No	No	No
Jin 2016 [147]	Classification	PP	Non-medical	Improved random forest	1 image, 1 patient	No	No	Yes
Kashala 2020 [148]	Classification	A	Non-medical	FireNet module in SqueezeNet and obtained FCN as well-developed new particle swarm optimization called NPSO	LiTS 2017: 70 patients; 3D-IRCADb: cross-validation	No	No	Yes
Khalili et al 2020 [149]	Classification	A	Non-medical	CNN	Validation: 2 patients; test: 2 × 10 patients	No	Yes	Yes
Kumar 2013 [150]	Classification	A	Non-medical	Probabilistic neural network	150 images	No	No	No
Kutlu 2019 [151]	Classification	A	Non-medical	CNN with alexnet architecture, DWT (Discrete Wavelet Transform) and Long short-terms memory networks	30% of data for test	No	No	No
Yasaka et al 2018 [152]	Classification	A	Medical	CNN	100 patients/image sets	Yes	Yes	No
Xin et al 2020 [56]	Classification	A	Non-medical	CNN	32 patients, 643 slices containing lesions	No	No	No
Sreeja and Hariharan 2017 [153]	Classification	PP	Non-medical	SVM + Naive Bayes classifier	Not specified	No	No	No
Shi et al 2020 [154]	Classification	A	Medical	CNN	One per lesion	No	No	No
Selvathi et al 2013 [64]	Classification	PP	Non-medical	ELM + FCMC	Not specified	No	No	No
Sayed 2016 [65]	Classification	PP	Non-medical	Fuzzy clustering + GWO (liver and liver lesion segmentation); SVM (liver disease classification: benign/malignant)	Not provided	No	No	No
Romero et al 2019 [155]	Classification	PP	Non-medical	CNN (FCN × 2)	(1) 46 lesions for validation, 46 lesions for testing; (2) not specified	No	No	Yes
Renukadevi and Karunakaran 2020 [156]	Classification	A	Non-medical	DBN + GOA	Not specified	Yes	No	Yes
Rajathi 2019 [157]	Classification	A	Non-medical	WOA-SA + SVM + k-NN + RFC	21 patients	No	No	No
Raj 2016 [68]	Classification	PP	Non-medical	SVM	Not specified	No	No	No
Ponnoprat et al 2020 [71]	Classification	A	Non-medical	U-Net for segmentation + CRF for post-processing + SVM for classification (w GHI kernel)	17 patients, 2042 images	No	No	No
Peng et al 2020 [158]	Classification	A	Medical	CNN (ResNet50)	ZHHAJU: 89; SYUCC: 138 patients	Yes	No	No
Özyurt et al 2019 [159]	Classification	A	Non-medical	CNN	34	No	No	No
Ouhmich et al 2019 [72]	Classification	A	Medical	U-Net	Not specified	No	No	No
Nayak et al 2019 [74]	Classification	A	Medical	Segmentation: region-growing; classification: SVM	Not specified	No	No	Yes
Mukherjee et al 2013 [75]	Classification	PP	Non-medical	SVM + PCA	Not specified	No	No	No
Mofrad et al 2014 [78]	Classification	A	Medical	SVM (classification), k-NN (classification)	1 patient	No	No	No
Mala et al 2015 [160]	Classification	A	Non-medical	PNN, LVQ, BPN	20 patients, ca. 20 images per patient	No	No	No
Maaref et al 2020 [161]	Classification	A	Medical	2D CNN (Inception-Net, modified)	CLASSIFICATION: 20 patients for validation, 41 for testing; PREDICTION: 12 patients for validation, 24 for testing	No	No	No
Selvaraj 2013 [82]	Classification	PP	Non-medical	FCM (lesion segmentation) + BPSO (feature selection) + PNN (classification)	15 images	No	No	No
Li et al 2019 [162]	Classification	PP	Non-medical	BPN (+ PCA preprocessing)	57 (tenfold cross-validation)	No	No	No
Liang et al 2018 [163]	Classification	PP	Non-medical	CNN (ResNet w/ global and local pathways—for segmentation) + SVM (classification)	(1) Validation: 115, test: 96; (2) validation: 93, test: 110	No	No	No
Liang et al 2018 [163]	Classification	PP	Non-medical	CNN (ResNet w/ global and local pathways w/ bi-directional long short-term memory—for segmentation) + SVM (classification)	(1) Validation: 115, test: 96; (2) validation: 93, test: 110	No	No	No
Xin et al 2020 [56]	Classification	A	Medical	CNN	643 slices for test	No	No	0
Thuring et al 2020 [164]	Classification	A	Medical	Random forest and CNN	70 patients	No	Yes	Yes
Wang et al 2021 [165]	Classification	A	Medical	Nodule Net and HCCNet	385 from same hospital, external test set with 556 patients	Yes	Yes	0
Wang et al 2020 [166]	Classification	A	Non-medical	CNN (Siamese cross contrast neural network)	67 patients	No	No	0
Xu et al 2021 [167]	Classification	A	Medical	Random forest	tenfold cross-validation	No	No reference	0
Zhang et al 2020 [168]	Classification	A	Medical	GLM	57	No	No	0
Zhou et al 2021 [116]	Classification	A	Medical	CNN	1/4 of lesion was used for test set	No	No	0
Giannini et al 2020 [169]	Classification	A	Medical	Gaussian Naive Bayes classifier	10 patients, 33 tumors/metastases	No	No	No
Homayounieh et al 2020 [170]	Classification	A	Medical	Random forest	103 patients w benign (60/103) or malignant (43/103) tumors	No	No	No
Mao et al 2020 [171]	Classification	A	Medical	Gradient boosting (XGBoost)	60 patients	No	No	No
Mokrane et al 2020 [172]	Classification	A	Medical	Random forest	36 patients	Yes	No reference	No
Budai et al 2020 [173]	Miscellaneous	A	Medical	RF and SVM, K-means clustering	Independent validation dataset from > Sliver07(20 dataset), > MICCAI 2017 (LiTS) 131 scans	No	No reference	Yes
Choi et al 2018 [33]	Miscellaneous	A	Medical	CNN	150 images	Yes	Yes	No
Huo et al 2019 [174]	Miscellaneous	A	Medical	DCNN and morphological operation for attenuation and SS-Net (a DCNN model)	Not specified	Yes	Yes	Yes
Kayaalti et al 2014 [175]	Miscellaneous	A	Non-medical	SVM and K-nearest neighbors for classifying the images		No	No	No
Yasaka et al 2018 [176]	Miscellaneous	A	Medical	CNN	100 portal phase images from 100 patients	No	Yes	No
Son et al 2020 [177]	Miscellaneous	A	Medical	U-net	Not specified	No	Yes	No
Yin et al 2021 [178]	Miscellaneous	A	Medical	CNN	fivefold cross-validation	No	No	0
Ahmadi et al 2016 [179]	Miscellaneous	A	Medical	FCM and GA	Test dataset 1: 150 patients, test dataset 2: 50 patients	No	No	No
Ben-Cohen et al 2018 [180]	Miscellaneous	PP	Non-medical	U-net base—using unlabeled data features in supervised network	Test set 1: 421 patients. Test set 2: 298 (other institutions). Test set 3: 172 patients (from tertiary referral hospitals	No	No	No
Bevilacqua et al 2017 [27]	Miscellaneous	PP	Non-medical	ANN classifier by using mono-objective genetic algorithm (GA)	Not mentioned	Yes	No reference	No
Conze et al 2017 [181]	Miscellaneous	A	Medical	Scale adaptive super voxel-based random forests	Not specified	No	No reference	No
Fu et al 2019 [6]	Miscellaneous	A	Non-medical	U net-with multi stream feature fusion and multi scale dilated convolution, author called it M-Net	Not specified	No	No	No
Gensure et al 2012 [182]	Miscellaneous	A	Medical	SVM	Not provided	No	No	No
Huang et al 2018 [183]	Miscellaneous	A	Medical	3d U-Net	Not specified	Yes	Yes	Yes
Kumar et al 2016 [50]	Miscellaneous	A	Medical	SVM, weighted nearest neighbor	50 CT images were used from ImageCLEF 2014 with tenfold cross-validation	No	No	Yes
Zhang et al 2018 [52]	Miscellaneous	A	Non-medical	Fuzzy connectedness (fuzzy logic)	(1) VascuSynth: not eligible; (2) 3D-IRCADb: not provided; (3) Sliver07: 10 patients	Yes	No	Yes
Zeng et al 2016 [184]	Miscellaneous	A	Medical	ELM	100,000 images in total (training + testing data)	No	No	No
Yu et al 2019 [185]	Miscellaneous	PP	Non-medical	CNN	6 cases (+ 3 for validation); slices per case range: 135–500	No	No	No
Yang et al 2012 [186]	Miscellaneous	A	Medical	k-means	Not specified	No	No	No
Xin et al 2020 [56]	Miscellaneous	A	Non-medical	CNN	32 patients, 643 slices containing lesions	No	No	No
Wang et al 2018 [59]	Miscellaneous	PP	Non-medical	BoVW (K-CP with multilinear OMP, K-nearest neighbor)	Leave-on-out cross-validation is used for testing	No	No	No
Taghavi et al 2021 [9]	Miscellaneous	A	Medical	Random forest	21 patients	No	Yes	No
Ponnoprat et al 2020 [71]	Miscellaneous	A	Non-medical	U-Net for segmentation + CRF for post-processing + SVM for classification (w GHI kernel)	17 patients, 2042 images	No	No	No
Maaref et al 2020 [161]	Miscellaneous	A	Medical	2D CNN (Inception-Net, modified)	CLASSIFICATION: 20 patients for validation, 41 for testing; PREDICTION: 12 patients for validation, 24 for testing	No	No	No
Wang et al 2017 [187]	Miscellaneous	A	Non-medical	BoVW (sparse codebook-based feature representation)	(leave-one-out cross validation)	No	No	No
Li et al 2020 [188]	Miscellaneous	A	Medical	ResNet	69 patients, 3 images per patient (fivefold cross-validation)	No	No	No
Lee et al 2020 [8]	Miscellaneous	A	Non-medical	CNN + RFC and CNN + LRC	606 patients	No	No	No
Sun et al 2020 [189]	Miscellaneous	PP	Non-medical	SVM	34 labeled CT	No	No	0
Thuring et al 2020 [164]	Miscellaneous	A	Medical	Random Forrest & CNN	70 patients	No	Yes	Yes
Wang et al 2020 [166]	Miscellaneous	A	Non-medical	CNN (residual CNN)	70slices (17 patients)	No	No	0
Xu et al 2020 [190]	Miscellaneous	PP	Non-medical	CNN (Deep neural network)	20 from 3dIRCADb	Yes	No reference	Yes
Yang et al 2021 [191]	Miscellaneous	A	Non-medical	CNN (v-net)	8 CT	No	No	Yes
Yoshinobu et al 2020 [192]	Miscellaneous	PP	Non-medical	CNN (Deep CNN)	32 cases	No	No	0
Zhang et al 2020 [124]	Miscellaneous	A	Medical	CNN (DenseNet)	From multicenter data from 3 hospitals	Yes	Yes	Yes
Gu et al 2020 [193]	Miscellaneous	PP	Non-medical	CNN + ResNet	1 patient	No	No	No
Kobe et al 2021 [194]	Miscellaneous	A	Medical	ANN	21 metastases/lesions	No	No reference	No
Li et al 2022 [195]	Miscellaneous	A	Medical	CNN (DenseNet)	244 patients	No	Yes	Yes

We encountered studies with 19 different aims. To make comparison and discussion more feasible, we divided these studies into five groups according to study aim: (1) liver segmentation; (2) lesion segmentation; (3) lesion detection; (4) classification of liver or liver lesions; (5) miscellaneous/other. Aims are illustrated in electronic supplementary material. There is some overlap in the groups due to several studies having multiple aims. Detailed characteristics of included studies are given in supplementary tables.

### Liver segmentation

The aim of liver segmentation was the primary or secondary study aim in eighty-four of the included studies. Of those, fifty-one are journal articles [20, 24, 29–35, 38–41, 43–47, 49, 55–58, 62, 63, 65, 68, 70–79, 81, 84–87, 89, 91, 93–95, 97, 98, 196, 197], and 33 are proceeding papers [19, 21–23, 25, 26, 36, 37, 42, 48, 51, 53, 54, 59–61, 64, 66, 67, 69, 80, 82, 83, 88, 90, 92, 96, 99, 100, 103, 198]. The liver segmentation was done from the CT as a whole liver, not the clinical segmentation, e.g., Couinaud segments of the liver. Overall, this group of studies has contributed considerably with technically sound methods and experimented with various subdomains of ML, especially DL.

The quality of many recent studies has improved using external validation method to provide better generalizability. Though comparing directly with human experts is preferred, only eleven studies were found to do so.

The study group gives insinuation of obtaining labeled medical data which is challenging, as two-thirds of studies used datasets open for public use for training or testing their ML model. The dataset from LiTS 2017, which was the most frequently used, included 131 patients in their test set [199].

The attempt of transparency in reporting models’ performance was seen in many studies, though out of eighty-seven studies, only 11 reported their results with confidence interval or standard error; thus, further analyses of the result were not feasible in the group.

DICE score was used in most studies in this group to describe the model’s ability to predict which pixel contains the liver. The highest DICE reported was a score of 0.9851 [41], and the lowest score was 0.75 [94]. Other measures to describe the model’s performance were scattered, including AUC-ROC and accuracy (Table 2). Dong et al also reported a DICE of 0.92 and an accuracy of 0.9722 from their study, and the AUC of 0.96. References of studies in the group are in Table 3.

**Table 2:** Definition of performance and outcome measures

Segmentation	refers to a pixel-wise classification of images throughout this review. This is the standard meaning of “segmentation” of images in data science and engineering. It is not to be confused with anatomical segmentation like the Coineaud segmentation of liver lobes, commonly used for clinical segmentation of the liver according to the portal blood supply (19)
DICE	describes the percentage of overlap between the predicted and the observed/”correct” labeled area in an image (often labeled by a human radiologist), where 1.0/100% means a perfect overlap between predicted and correct segmentation
Accuracy	related to image segmentation in engineering is a measure describing how many pixels are correctly classified—1.0/100% being perfect. However, accuracy can be misleading in cases where a class is in very few pixels; for instance, a small tumor could be only in 2% of the image—and a model predicting that there are 0% tumors would still have an accuracy of 98%. Therefore, if only accuracy is reported for performance, a measure of class balance might be relevant to the readers' understanding
Precision and Recall	Precision is the number of relevant observations by a model divided by the total number of observations made by the model. For instance, if a model marks 100 pixels as tumor tissue and 40 are tumor tissue, the precision is 40%/0.4. Precision is the same as positive predictive value (PPV). Recall is the number of relevant observations divided by the total number of actual cases, e.g., if an image contains 100 pixels with actual tumor tissue, and the model observes 80 of them, the model has a recall of 80%/0.8. In binary classification cases, recall is the same as sensitivity, hit rate, and true positive rate
Volume Overlap Error (VOE)	gives a measure of the difference between actual area and predicted area. It functions as a combined score of both false positives and negatives ${\varvec{V}}{\varvec{O}}{\varvec{E}}\left({{\varvec{U}}}_{1},{{\varvec{U}}}_{2}\right)=100\times \boldsymbol{ }(1-\boldsymbol{ }\frac{{{\varvec{U}}}_{1}\cap \boldsymbol{ }{{\varvec{U}}}_{2}}{{{\varvec{U}}}_{1}\cup \boldsymbol{ }{{\varvec{U}}}_{2}})$ where U_1_ and U_2_ are true and predicted values, respectively. Optimal scores are as low as possible, 0 being the perfect score (20)
IoU / Jaccard Index	The intersection over union (IoU), is a measure that quantifies the percentage of overlap between prediction and observed/true output, much like the DICE coefficient. IoU measures the overlapping pixels between true and predicted segmentation and divides it by the total number of pixels either of them has marked as a pixel of interest. A perfect score would be 100%/1.0. This measure is also referred to as the Jaccard Index
Ground truth	refers to the label for anatomical structures in CT images given by a clinician or radiologist. What kind of expert and level of experience is often specified in each specific study
CNN	refers to Convolutional Neural Network – a deep learning model based on vector calculations used in image recognition and processing pixel data

**Table 3:** References of studies in each category according to characteristics

Characteristics of studies	Liver segmentation	Lesion segmentation	Lesion detection	Classification of liver or lesions	Miscellaneous
Journal article	51 studies [20, 24, 29–35, 38–41, 43–47, 49, 55–58, 62, 63, 65, 68, 70–79, 81, 84–87, 89, 91, 93–95, 97, 98, 196, 197]	36 studies [24, 29, 31, 32, 38, 46, 47, 55, 56, 62, 72, 78, 84, 91, 93, 94, 97, 98, 111, 115, 117, 118, 120, 122, 124, 125, 130, 133–135, 137, 138, 140, 199]	5 studies [101, 106, 111, 115, 202]	34 studies [56, 71, 72, 74, 78, 141–146, 148–152, 154, 156–161, 164–172, 202, 203]	29 studies [6, 8, 9, 33, 50, 52, 56, 71, 161, 164, 173–179, 181–184, 186–188, 191, 194, 195, 205, 206]
Proceeding papers	33 studies [19, 21–23, 25, 26, 36, 37, 42, 48, 51, 53, 54, 59–61, 64, 66, 67, 69, 80, 82, 83, 88, 90, 92, 96, 99, 100, 103, 198]	24 studies [22, 37, 42, 64, 65, 68, 82, 88, 92, 96, 99, 103, 108, 121, 124, 126–129, 131, 132, 136, 139, 200]	15 studies [23, 26, 27, 87, 102, 104, 105, 107–110, 112–114, 119]	13 studies [27, 64, 65, 68, 75, 82, 119, 147, 153, 155, 162, 163, 204]	8 studies [27, 180, 185, 189, 190, 192, 193, 207]
ML to human expert	10 studies [20, 23, 24, 32, 33, 35, 58, 76, 81, 98]	6 studies [24, 32, 76, 98, 134, 140]	2 studies [23, 106]	5 studies [143, 149, 152, 164, 165]	9 studies [9, 33, 164, 174, 176, 177, 183, 195, 206]
Using public datasets	57 studies [19–21, 23–26, 28–31, 34–37, 39, 41, 43, 46, 49, 51, 54, 55, 57, 59, 60, 62, 66, 69, 70, 74, 76, 77, 79–81, 83–86, 88, 89, 91–96, 98–100, 103, 196, 198]	38 studies [24, 29, 31, 37, 38, 46, 47, 55, 62, 76, 79, 84, 88, 89, 91–94, 96–99, 118, 122, 124–129, 132, 136–139, 200]	8 studies [23, 26, 101, 104, 106, 108, 112, 119]	12 studies [74, 119, 141–143, 145, 147–149, 155, 156, 164]	10 studies [20, 50, 52, 173, 174, 183, 190, 191, 195, 206]
Reporting of standard error	11 studies [20, 21, 27, 31, 34, 39, 49, 88, 90, 196, 197]	7 studies [29, 31, 47, 88, 115, 120, 122]	2 studies [27, 115]	3 studies [27, 143, 165]	8 studies [33, 50, 173, 178, 179, 181, 190, 205]
Reporting of DICE score	55 studies [20–22, 24–26, 29–34, 37, 38, 42, 46–48, 51, 54–57, 59–62, 65, 66, 69–74, 77, 79, 85, 88–92, 94–96, 98–100, 103, 196, 198]	42 studies [22, 24, 29, 31, 32, 37, 38, 42, 46, 47, 55, 56, 62, 72, 76, 79, 84, 88, 89, 91, 92, 94, 96–99, 103, 115, 117, 120, 122, 124, 125, 127, 129, 131, 137, 139, 200]	4 studies [27, 106, 115, 202]	10 studies [56, 65, 72, 74, 144, 145, 155, 165–167]	12 studies [52, 56, 71, 179–183, 185, 189–191]
Reporting of accuracy	13 studies [19, 35, 36, 38, 53, 63–65, 74, 76, 82, 93, 94]	8 studies [42, 64, 65, 94, 118, 130, 138]	4 studies [23, 102, 108, 119]	31 studies [27, 64, 65, 71, 74, 75, 82, 119, 142–145, 151, 153–165, 168, 202–204]	19 studies [6, 27, 33, 50, 52, 71, 161, 164, 174, 175, 178, 182, 184, 187–191, 193]
Reporting of AUC	3 studies [23, 38, 94]	4 studies [38, 94, 111, 115]	3 studies [23, 111, 115]	16 studies [56, 74, 75, 143, 152, 154–156, 158, 161, 164, 167, 168, 170–172]	12 studies [8, 9, 33, 161, 173, 176–178, 188, 194, 195, 205]
Reporting of precision	8 studies [23, 26, 34, 63, 74, 87, 95, 98]	5 studies [32, 56, 98, 111, 128]	9 studies [23, 87, 105, 108, 111, 113, 114, 202]	13 studies [56, 65, 74, 119, 143, 150, 153–156, 160, 165, 170]	5 studies [6, 173, 186, 192, 207]
Reporting of VOE	17 studies [21, 30–32, 35, 36, 39, 46–48, 89, 91, 97, 100, 103, 196, 197]	24 studies [31, 32, 46, 47, 55, 62, 89, 91, 97, 103, 111, 120, 122, 123, 125–127, 129, 132, 136, 137, 201]	Not available	Not available	1 study [27]
External validation	32 studies [20, 21, 24–27, 30–33, 37, 39–45, 47, 48, 62, 76, 81, 84, 86, 88, 89, 91, 92, 94, 98, 103]	26 studies [24, 31, 32, 37, 42, 47, 62, 76, 84, 88, 89, 91, 92, 94, 98, 103, 115, 118, 120, 122–125, 132, 136, 140]	4 studies [26, 27, 111, 115]	8 studies [27, 146, 151, 152, 156, 158, 165, 172]	7 studies [27, 33, 52, 174, 183, 190, 206]

### Lesion segmentation

This group of studies performed segmentation of liver lesions from CT images with ML. The model’s goal was the highest possible truthfulness of segmented lesions compared to ground truth. Sixty studies had lesion segmentation as a primary or secondary study aim. Thirty-six are journal articles [24, 29, 31, 32, 38, 46, 47, 55, 56, 62, 72, 78, 84, 91, 93, 94, 97, 98, 102, 111, 115, 117, 118, 122, 124, 125, 130, 133–135, 137, 138, 140, 201], and twenty-four [22, 37, 42, 64, 65, 68, 82, 88, 92, 96, 99, 103, 108, 121, 124, 126–129, 131, 132, 136, 139, 200] are proceedings papers.

Several models have shown remarkable segmenting ability for predicted lesions larger than 2 cm in diameter, while almost every model is still struggling to segment lesion size less than 1 cm in diameter. However, this is comparable with clinicians in the clinical setting. Another limitation for the model to predict the lesion was quality of CT images. Several more recent studies used voxel-wise (3D pixels) classification. This could use more available information and give output in 3D to improve performance.

Validation of the model with external validation and ML to humans is improving for this group, and twenty-six studies have used external validation. Only six studies have compared their model with human experts.

More than half of the studies have reported performance in a DICE score in this group. The score range was seen skewed in different studies with the range of 0.44–0.96; a selection of lesion size played a key role here for higher performance or higher DICE score. Another informative measure called Volume Overlap Error (VOE) gives the difference between predicted and ground truth in an area. Thus, 0 is the optimal score. Twenty-two studies reported VOE, with a 0.01–0.46 mm range. Other measures were dispersed in different studies, including accuracy, AUC, precision, or PPV. Few studies have reported their performance with confidence intervals or standard errors—references of studies in the group in Table 3.

### Lesion detection

Twenty studies had lesion detection as a primary or secondary study aim. This involves simply detecting if lesions are present in a CT image. Fifteen of them are proceedings papers [23, 26, 27, 87, 102, 104, 105, 107–110, 112–114, 119], and five are journal articles [101, 106, 111, 115, 202].

Several newer studies have detected lesions before segmentation of the lesions or diagnosis of the lesions with ML from CT liver images but have not reported performance of the lesion detection task of the model; thus, this group is smaller.

External validation was reported only in four studies. Most studies acquired their training data from local hospitals, and only eight studies have used data sets open for public use. DL was the choice of a subdomain of ML for this group.

Reporting of performance was seen as transparent and detailed in newer studies in all groups. In this group, performance was primarily reported in accuracy and precision, but five studies reported only false positives and true positive rate [26, 87, 101, 104, 115]. Two studies presented its result with a confidence interval or standard error. It is worth mentioning that the study reporting the best precision only performed internal validation on the relatively small, public dataset 3D-IRCADb—references of studies in the group in Table 3.

### Classification of liver or lesions

Included studies in this group classifying the type and severity of lesions or tumors, grading hepatocellular carcinoma (HCC), and differentiating between HCC, hemangioma, and metastases. Most studies differed only between two categories, such as classifying tumors as either benign or malign. Forty-seven studies had the classification of liver or liver lesions as a study aim. Thirty-four of them journal articles [56, 71, 72, 74, 78, 141–146, 148–152, 154, 156–161, 164–172, 202, 203], and thirteen are proceedings papers [27, 64, 65, 68, 75, 82, 119, 147, 153, 155, 162, 163, 204]. For classification of liver or liver lesions, traditional machine learning, e.g., support vector machines and random forest models, and deep learning models were commonly used. 

Nine studies compared their model performance directly to one or more clinicians in a competition-based comparison. Only 12 studies have used datasets open for public validation, and even fewer are needed for training purposes.

Accuracy was a method of choice to present the performance in this group; thirty-one studies reported accuracy, with a range of 0.76–0.99. Sixteen studies reported AUC, with a range of 0.68–0.97. Precision was reported in fourteen studies. The precision range was 0.82–1.00. Note that both Sreeja et al and Romero et al reported a perfect precision of 1.0, which Sreeja et al commented was possible due to the small size of their data set [153, 155]. Only three studies presented their result with a confidence interval—references of studies in the group are in Table 3.

### Other/miscellaneous

The last and most diverse category we found eligible to compare was miscellaneous, including 29 journal article [6, 8, 9, 33, 50, 52, 56, 71, 161, 164, 173–179, 181–184, 186–188, 191, 194, 195, 205, 206] and 8 proceeding paper [27, 180, 185, 189, 190, 192, 193, 207] total thirty-seven studies. The aims of the studies are clinical-oriented.

Seven studies have performed liver fibrosis staging [33, 173–178] according to “Metavir” or “Fibrosis-4” classification [208, 209]. Four compared algorithms performance with human expert while two studies performed external validation. Only two studies used public dataset for liver segmenting purpose; however, private datasets were used for fibrosis staging training and validation purpose in all the seven studies. ML method like SVM, k-nearest neighbor were used traditionally but in the recent studies, CNN-based systems using different classifier to extract the feature from the liver image are gaining more attention. Jung et al used liver and spleen volumetric indices and perform the pathologic liver fibrosis staging with CNN [177]. Comparison of ML algorithm to 3 radiologists’ assessment of liver fibrosis staging was performed with more accurate result in ML group [33].

Six studies segmented blood vessels in the liver from CT images, including portal and liver veins [52, 179, 183–185, 191]. Twelve studies reported a DICE score with a range of 0.68–0.98. The four studies reported accuracy with a range of 0.91–0.98, with a mean of 0.96 and a median of 0.97. Five studies stated that they externally validated their model.

Five retrieved focal liver lesion images as a study aim [50, 186, 187, 192, 206]. These studies showed how models could improve clinical workflow by retrieving similar cases in medical records, including earlier expert opinions.

Two studies, published as journal articles, predicted liver metastases within colorectal cancer patients [8, 9]. They reported AUC equal to 0.86 ± 0.01(12) and 0.747 ± 0.036.

One study focused on the segmentation of bile ducts and stones in the intrahepatic bile duct—hepatolith and reported DICE of 0.90 and 0.71 for bile duct and hepatolith segmentation, respectively [6].

Three study focused on response evaluation after chemotherapy or radio-embolization of malignant liver lesions using texture analysis [161, 181, 182]. They compared texture analysis predictions with survival and serologic response and reported an accuracy of 0.97, sensitivity of 0.93, and specificity of 1.0. This was after training on sixty-two patients and testing using cross-validation.

Two recent studies have predicted liver reserve function using Child–Pugh classification [164, 189] and Thuring et al have compared the results from their ML model with results from clinicians. Prediction of Child–Pugh accuracy was 53%, classification of Child–Pugh A vs B: accuracy was 78%, sensitivity 81%, specificity 70%, and AUC 0.80. Wang et al had preoperatively predicted early recurrence in HCC. One study has predicted overall survival of patients with unresectable HCC treated by transarterial chemoembolization [176]. This study also presented fusion of clinical data with ML model. References of studies in the group in Table 3.

## Discussion

We found that ML is applied to liver CT imaging for various clinical oriented aims and covering a broad spectrum of applications.

At least one-third of studies were documented to perform very accurately on reliable, but small data. Unfortunately, reporting of performance was seldom appropriate due to lack of details. To our knowledge, there exists no standardized form of presenting results for machine learning models applied to medical imaging.

Several studies reported models that were close to clinical application. However, clinical validation with thorough documentation of both model and data (training and validation) to assess quality and generalizability were lacking. Evaluation of the model by only analysis of a result parameters would be imperfect [210].

**Fig. 2:**
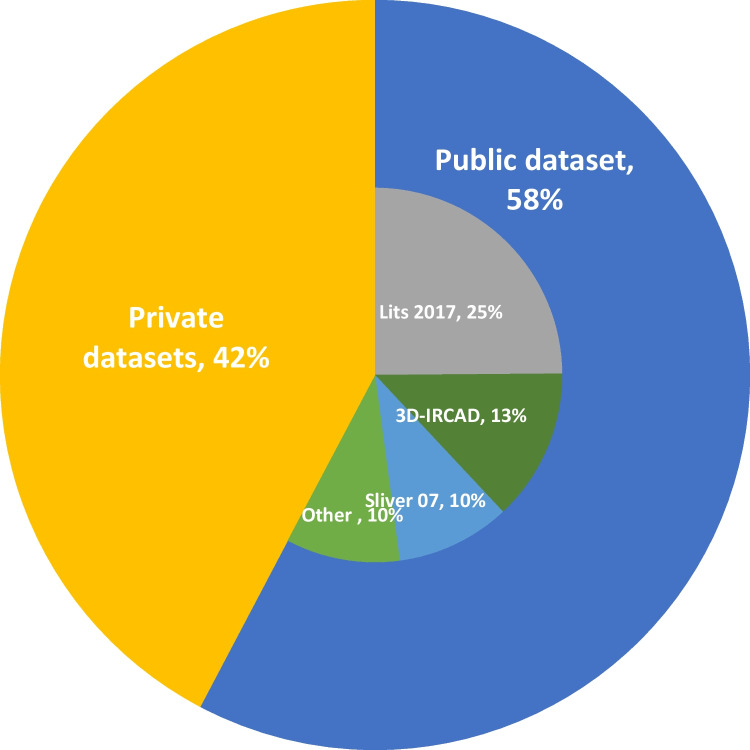
Distribution of used dataset in the model for training and validation purpose. Publicly available datasets include Lits 2017, 3D-Ircadb, Sliver 2017 and other, while private dataset were mostly collected from local hospitals

Almost all studies that performed segmentation of liver structures from the CT images of the abdomen used deep learning models, mainly the subtype CNN. Open-access datasets and competitions like LiTS 2017 contribute substantially to the development of ML applied to liver imaging, as more than 280 studies report their model performance in a standardized format, and the competition is still ongoing with cumulative comparison. U-Net a sub domain of CNN is used by many participants and have shown promising result. The distribution of sources of dataset used by studies included in this review is illustrated in Fig. 2. The use of complex models and targeting for complex aims like automatic liver fibrosis staging, treatment response evaluation, prediction of occurrence of liver metastases, and liver blood vessels segmentation for traditional anatomical landmarks, e.g., Coineaud classification, are getting more common and may herald a maturing process in the field.

ML systems showed promising results on retrospective data for several tasks related to CT imaging, as some segmentation studies reported models with more than 98% ability to predict which pixels or voxels contained liver in abdominal CT scans. Further, several studies reported 95% performance compared to ground truth for liver or liver lesions classification. In recent years, identified studies have used ML for prediction of occurrence or treatment effect of liver metastases, liver vessel segmentation, and evaluation of treatment effect on liver malignancy. These showed results around 70–80% of ground truth.

Other applications such as classification of liver fibrosis stage and prediction of benign or malign lesions showed promising results and potential for the high impact of ML in future routine clinical practice.

Reporting of model performance should give in the state-of-the-art visualization methods, e.g., confusion matrix. In the studies like segmentation task, measuring parameter like mean surface distance with standard error should be reported to get overall transparency of the model performance [116]. Sixty-two studies identified in this review have such breach in reporting of model performance. This makes it difficult to get a good overall understanding of the field, especially for clinicians. We encourage the readers to assess such results with caution.

Further, reporting of standard error and confidence intervals was often lacking. We recommend that it should be reported by default. This problem was also seen in other applications of ML to medical images, and we concur with the need for reporting standards for medical application as stated by Aggarwal et al [10].

There are potentially many applications of ML in liver CT imaging have been identified thorough this review, especially in the miscellaneous group aims are clinically derived, while segmenting of liver and its lesions could implement as diagnostic and treatment planning tool. Studies in classification group could serve diagnosis of different lesions, e.g., different types of malign and benign tumors, or severity of the liver cirrhosis. Despite the promising performance reported in many studies, clinical applications of ML in liver CT imaging have to pass through the corridor of clinical validation and clinical trials [210].

**Fig. 3:**
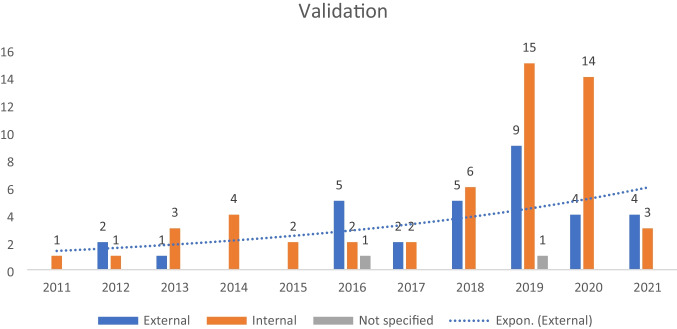
Bar-chart categorize by validation method in timeline. An increasing trend of external validation from 2011 to 2021 are demonstrated in dotted line

The main issues identified in the literature were limited access to high-quality data and lack of clinical validation. External validation is becoming more popular among developers, illustrated in Fig. 3, but it is insufficient to qualify for medical application. There is an urgent need for a shift in focus towards clinical validation in this field. Scholars should perform feasibility studies in clinical routine, and design and carry out prospective studies to validate the performance of ML tools in realistic clinical settings. Developers should seek to collaborate with clinicians in this process. Strength and weakness of the study as well future perspective is given in the supplementary material.

## Conclusion

We found reports of many ML applications to liver CT images in the literature, including automatic liver and lesion segmentation, lesion detection, liver or lesion classification, liver vessel segmentation including bile ducts, fibrosis staging, metastasis prediction, and evaluation of chemotherapy as treatment of hepatocellular carcinoma and retrieval of relevant liver lesions from other similar cases. Several were documented to perform very accurately on reliable but small data. Deep learning models and classification models of ML were commonly used. However, presenting the result of studies is not standardized in the literature. Some studies were close to reporting sufficient details on clinical relevance, data characteristics and quality, algorithm characteristics and bias, and performance measures on external data to be considered ready for clinical use. Further prospective, clinical studies are recommended, and the need for a more interactive technological and medical research is evident to achieve a secure clinical use of ML methodology in this field.

### Supplementary Information

Below is the link to the electronic supplementary material.

Supplementary file1 (PDF 176 KB)

## Data Availability

Custom code or mathematical algorithms were not used and do not play any role in our conclusion.

## Abbreviations

3D RA U-Net: 3D hybrid residual attention U-shaped neural network
A: Article
ACM: Auto-context model
AHC Blocks: Attention hybrid connection blocks
ANN: Artificial neural network
ASM: Active shape model
BPSO: Binary particle swarm optimization
CDNN: Convolutional—deconvolutional neural network
CEDCNN: Cascade encoder-decoder CNN
CENet: Contour embedded neural network
CNN: Convolutional neural network
CRF: Conditional random field
CT: Computed tomography
DBN-DNN: Deep belief network-deep neural network
DCT: Discrete cosine transforms
DL: Deep learning
DLA: Deep learning algorithm
DLO: Dice loss
DResU-Net: Deep residual U-net
DRL: Deep reinforcement learning
ELM: Extreme learning machine
FCMC: Fuzzy C-means clustering
FCN: Fully convolutional neural network
FCNN: Fully convolutional neural network
GAN: Generative adversarial network
GDL: Generalized dice loss
GLC U-Net: Global and local contexts composition U-shaped neural network
GTL: Generalized Teverskry loss
GWO: Grey wolf optimization
HCC: Hepatocellular carcinom
HCC: Hepatocellular carcinoma
HDCNN: Hybridized fully convolutional neural network
k-NN: k-nearest neighbor
ML: Machine learning
MOGA: Multi objective genetic algorithm
MPNet: Message passing neural network
MRF: Markov random field
MSCA: Mean-shift clustering algorithm
MW-U-Net: Modality weighted U-net
PCA: Principal component analysis
PNN: Probabilistic neural network
PP: Proceeding paper from conference
R-CNN: Region based convolutional neural network
RES-U-Net: Residual U-net
RFC: Random forest classifier
RL: Reinforcement learning
RPN: Region proposal network
SSD: Single-shot multibox detector
SSD: Support vector machine
TDP: Three-dimensional dual path multiscale convolutional neural network
TL: Teverskry loss
U-NET: U-shaped neural network (referes to the model architecture)
U-RES-Net: U-shaped residual neural network
VGG 16: Visual Geometry group 16 (Personal name of model named after a research group)
VOE: Volume overlap error

## Declarations

### Guarantor

The scientific guarantor of this publication is Keyur Radiya.

### Statistics and biometry

No complex statistical methods were necessary for this paper.

### Methodology

• Systematic review
