# Supplementary material for: Performance and clinical applicability of machine learning in liver computed tomography imaging: a systematic review
Source: Eur Radiol. 2023 May 12;33(10):6689–717. doi: 10.1007/s00330-023-09609-w (PMC10511359; doi:10.1007/s00330-023-09609-w)
Supplement: Supplementary file 1 — Supplementary file1 (PDF 176 KB) [file 330_2023_9609_MOESM1_ESM.pdf]

ELECTRONIC SUPPLEMENTARY MATERIAL

PERFORMANCE AND CLINICAL APPLICABILITY OF MACHINE LEARNING IN LIVER COMPUTED TOMOGRAPHY IMAGING: A SYSTEMATIC REVIEW

Method

The search string consisted of exploded MeSH-terms, Emtree-terms, and free text to find all studies containing the terms "Artificial intelligence" AND "Computed tomography" AND "liver" (or containing all possible synonyms of all three) in the title, abstract or keywords.

Search string

AI		CT		Liver
Artificial intelligence<br>• Computer Intelligence<br>• Machine Intelligence<br>• Computer Reasoning<br>• Computer Vision System<br>• computer System<br>• computer System<br>• computer vision<br>• Knowledge Acquisition<br>• Knowledge Representation<br>• machine learning<br>• deep learning<br>• cnn<br>• Convolutional neural network<br>• Convolutional network<br>• neural network<br>• Ai	AND	tomography, X-Ray computed<br>• Computer tomography<br>• x-ray<br>• computer assisted tomography<br>• ct x-ray<br>• Tomodensitometer<br>• computed x-ray tomography<br>• computed tomography,x-ray<br>• x-ray<br>• CAT Scan<br>• Cine-CT<br>• electron beam<br>• axial tomography	AND	Liver<br>• hepat*

After removing duplicates, all titles and abstracts were screened independently by the two first authors of this review (KR and HLJ), using the following criteria: Peer-reviewed studies reporting in English on the application of ML algorithms on original CT of human liver imaging data were

included. In addition, if fulfilling our research purpose, peer-reviewed full research papers published in proceedings from conferences were also included. In AI and computer science, such publications can be even more prestigious than journal articles, so we emphasize the importance of including them. Abstracts, pre-prints, reviews, and meta-analyses were excluded. Studies using animal or synthetic liver images were excluded. To ensure the quality of the search string, we searched within the retrieved titles for known relevant publications that had been identified from earlier reviews [11-13].

Important study quality characteristics

- Arguments for why the current methods should be updated
- Clinical success criteria and a description of how patient privacy has been ensured
- Sample size must be clearly stated, preferably with sample size calculation
- The investigated cohort should be described regarding representativeness, level of algorithm bias and calibration quality. Preferably including distribution of age, gender, relevant medical history, site, and inclusion/exclusion criteria
- Assessment of data quality, preferably with description of missing data and how it was dealt with
- Details on data preprocessing, postprocessing, benefits and potential risks associated with the chosen AI-technique, as well as details on training and validation scheme
- Thorough external validation details and assessment on the level generalizability, this includes measures taken to identify and reduce model overfitting, and measures to identify and prevent algorithmic bias
- Reports of operability and AI-human interaction in the clinical setting

The suggested list is comprehensive, and studies might be quite informative with minimal risk of bias, without meeting all requirements [17]. Yet, if a study followed only few of the characteristics, it was not considered well-documented for clinical use

Results

Aims

Liver segmentation	Lesion Detection	Lesion Segmentation	Classification	Miscellaneous
1.Liver segmentation	1.Lesion Detection	1.Lesion segmentation	1.Lesion Classification<br>2.Liver classification<br>3.Classification of HCC	1.Liver Volume<br>2.Bile duct segmentation<br>3.Detection of stone in bileduct<br>4.Response evaluation after chemomtherapy<br>5.Liver vasculature segmentation<br>6.Liver Data Augmentation technique<br>7.Retrieval of focal liver lesion images<br>8.Prediction of metastases within CRC patients<br>9.Prediction of treatment effect<br>10.Prediction of liver reserve function<br>11.Prediction of early recurrence of HCC after TACE treatment<br>12.Survival prediction<br>13.Liver fibrosis staging

19 different aims encountered in the included studies, and categorized in 5 groups; 1)Liver segmentation, 2) Lesion detection, 3)Lesion segmentation, 4)Classification, 5)Miscellaneous

Transparency

For tasks such as segmentation, accuracy was in many cases reported without further measures or tests. In an image of the liver, a mere 4% of the pixels might contain lesions. If the model predicts that there are no pixels containing lesions, it has an accuracy of 96%. If such a class imbalance is present, accuracy can be very misleading and insouciant. We encourage the readers to assess such results with caution.

Concept

Several measures can be applied to evaluate model performance. Methods like accuracy, precision, ROC /AUC, and DICE score, where both negative and positive predictive values are mandatory to calculate the scores, are in the direction of transparency to show the models'

performance. Reporting only positive or negative predictive values reduces the reliability of the model.

In medical images, interesting organs or findings containing areas or pixels are marked manually by clinicians or radiologists are called labeled data, and the location is marked and called ground truth. These data are used for training purposes in many models and for validation of the model to compare the predicted area or pixels to the ground truth.

Selection process

The search was conducted in two phases, one in October 2020 and one in September 2021. Our search retrieved 1334 studies, which were reduced to 808 after removing duplicates. Five hundred twenty-nine studies were excluded during the screening of abstracts using the eligibility criteria. Of these, 122 were excluded due to not using CT data as input for an ML model in their study. Further, ninety-one studies did not apply their model to liver data, fifty-three did not use any form of AI, and eighty-one studies did not contain any experimental or original data where AI was applied to CT liver imaging, such as reviews, case reports, editorials, surveys, or interviews, and was thus excluded. To be included, studies also had to use solely original CT images of in-vivo human liver, which excluded thirty-nine studies using non-human CT images, eighteen using not in-vivo images, and eight studies using synthetic CT images. Three (3) studies were excluded because they were not available in English, and eleven were excluded because they were not available in full text (nor upon request). Lastly, 108 studies were excluded due to the wrong outcome. Typically, these were computer science studies that applied ML models to multiple medical image modalities and organs to show an overall performance in the early development stages, without any details or focus on liver CT. Studies that were not accessible were sought through email or research-gate. Eighty studies were excluded during screening and data extraction. Finally, 191 studies were included in our study.

Discussion

Caution

Publicly available 3DIRCADb and LiTS 2017 datasets overlap, as some of the images are the same in both datasets. As a result, studies using one data set for training the model and the other for testing the model might have misleading results showing a better performance than what is the case. However, this mistake was only seen in three studies [22; 32; 113].

Future perspectives

Most of the studies used supervised learning to train their algorithms on small datasets. However, labeling large data sets is a time- and resource-consuming process, making it a common barrier in the training and development of ML models. Pursuing solutions using unsupervised or weakly supervised learning could make training more accessible and reliable in the future, as one could train on more extensive data without having to label all of it.

The amount and quality of data is the core element in ML models. Unfortunately, the availability of labeled data is minimal due to both technical and ethical issues. This should be an area of research focus where academical institutions should initiate and maintain such databases for further research.

Problems yet to be solved are data access, proper reporting of clinical validation, and user-friendly solutions for optimal ML-based decision support. We hope that regulating bodies will make data access easier. Universities and hospitals could contribute to creating databases available for research purposes. The safety and efficacy of medical tools are crucial to gain trust and acceptance among clinicians, and prospective clinical validation studies are considered the gold standard to achieve this. Thorough and transparent reporting is as essential for this as the actual validation. Further, ML will be implemented and used more if convenient, so documenting user-friendly ML applications could ensure a more significant impact.

We recommend that data scientists and engineers work with medical professionals to make their models properly validated and user-friendly in the future.

Strength and weakness of the study

In this study, we followed the PRISMA and PRISMA-P guidelines for systematic reviews, which included publishing the protocol in advance in PROSPERO. We have searched broadly for all possible relevant literature with an extensive search, including databases that are not exclusively for medical research. The inclusion of peer-reviewed proceedings papers is a strength of this study, as it is considered almost more prestigious than journal articles in engineering academia, especially related to ML.

On the other hand, the included studies were of varying quality with incoherent data reports and model performance. Meta-analysis was not possible because few studies reported standard error or confidence intervals. Incomplete performance reports combined with little or no

information on training and testing data can give an inaccurate and incomplete picture of performance and risk of bias in a model. Such studies should be read and interpreted accordingly.

A weakness of this study that cannot be ascribed to the included studies, is that we found it relevant to add some variables including “ML to human expert,” “use of public dataset”, “SD”, “RMSD”, “VOE”, “ASSD”, “RVD” and “Jaccard index” during data extraction that were not pre-defined in our protocol. In this process, we might have increased the risk of bias.
